# Efficient Production of Pyruvate from DL-Lactate by the Lactate-Utilizing Strain *Pseudomonas stutzeri* SDM

**DOI:** 10.1371/journal.pone.0040755

**Published:** 2012-07-09

**Authors:** Chao Gao, Jianhua Qiu, Cuiqing Ma, Ping Xu

**Affiliations:** 1 State Key Laboratory of Microbial Technology, Shandong University, Jinan, People’s Republic of China; 2 State Key Laboratory of Microbial Metabolism, Shanghai Jiao Tong University, Shanghai, People’s Republic of China; Massachusetts Institute of Technology, United States of America

## Abstract

**Background:**

The platform chemical lactate is currently produced mainly through the fermentation of sugars presented in biomass. Besides the synthesis of biodegradable polylactate, lactate is also viewed as a feedstock for the green chemistry of the future. Pyruvate, another important platform chemical, can be produced from lactate through biocatalysis.

**Methodology/Principal Findings:**

It was established that whole cells of *Pseudomonas stutzeri* SDM catalyze lactate oxidation with lactate-induced NAD-independent lactate dehydrogenases (iLDHs) through the inherent electron transfer chain. Unlike the lactate oxidation processes observed in previous reports, the mechanism underlying lactate oxidation described in the present study excluded the costliness of the cofactor regeneration step and production of the byproduct hydrogen peroxide.

**Conclusions/Significance:**

Biocatalysis conditions were optimized by using the cheap dl-lactate as the substrate and whole cells of the lactate-utilizing *P. stutzeri* SDM as catalyst. Under optimal conditions, the biocatalytic process produced pyruvate at a high concentration (48.4 g l^−1^) and a high yield (98%). The bioconversion system provides a promising alternative for the green production of pyruvate.

## Introduction

Lactic acid, the most important hydroxycarboxylic acid, is currently commercially produced by the fermentation of sugars present in biomass [1]–[3]. In addition to its use in the synthesis of biodegradable polymers [4], lactic acid can also be regarded as a feedstock for the green chemistry of the future [1]. For example, pyruvate, another important platform chemical, can be produced from lactate through direct oxidation [5]–[7]. Considering the significant difference in their prices, the production of pyruvate from lactate by catalysis is a valuable process.

**Figure 1:**
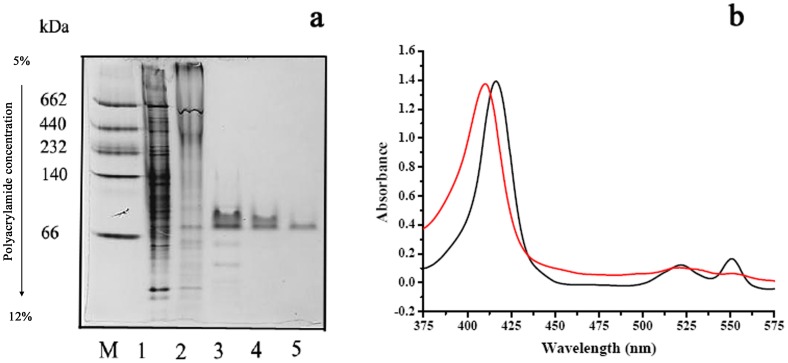
Involvement of cytochrome *c* in lactate oxidation of *P. stutzeri* SDM. **** **** **** **** **** (a) Native-PAGE of cytochrome *c* in *P. stutzeri* SDM. Lane M: molecular mass standards in kilodaltons (kDa) (GE Healthcare); lane 1: cell extract of *P. stutzeri* SDM; lane 2: membrane fractions of *P. stutzeri* SDM; lane 3: pooled fraction containing cytochrome *c* after DEAE-Sepharose; lanes 4 and 5: pooled fraction containing cytochrome *c* after DEAE-A25. (b) The absorption spectrum of cytochrome *c* in *P. stutzeri* SDM. Black line: reaction mixtures consisting of cytochrome *c*, crude extract of *P. stutzeri* SDM and dl-lactate. Red line: reaction mixtures consisting of cytochrome *c* and crude extract of *P. stutzeri* SDM.

**Table 1:** Effects of different respiratory chain depressors on the lactate oxidation activities.

Depressor	l-Lactate oxidation activity (U mg^−1^)	d-Lactate oxidation activity (U mg^−1^)
No inhibitor	0.170±0.004	0.091±0.004
Salicylhydroxamic acid (0.5 mM)	0.112±0.007	0.069±0.001
Diphenylamine (0.5 mM)	0.149±0.020	0.088±0.002
NaN_3_ (0.5 mM)	0.110±0.008	0.086±0.001
Antimycin A (0.1 mM)	0.022±0.002	0.036±0.001

Most of the reported chemical catalysts convert a major part of lactate to acetaldehyde and CO_2_ rather than pyruvate [7]. Indeed, there have been very few attempts to bring about the oxidative dehydrogenation of lactate through chemical catalysis [5], [6]. Biocatalysts could catalyze lactate to pyruvate under relatively mild conditions [1], [6]. Different enzymes, such as NAD-dependent lactate dehydrogenases (nLDHs) and lactate oxidase, have been employed in the biotechnological production of pyruvate from lactate. However, the costliness of cofactor NAD or the production of the byproduct hydrogen peroxide restricted the industrial application of nLDHs and lactate oxidase, respectively [1], [7].

**Figure 2:**
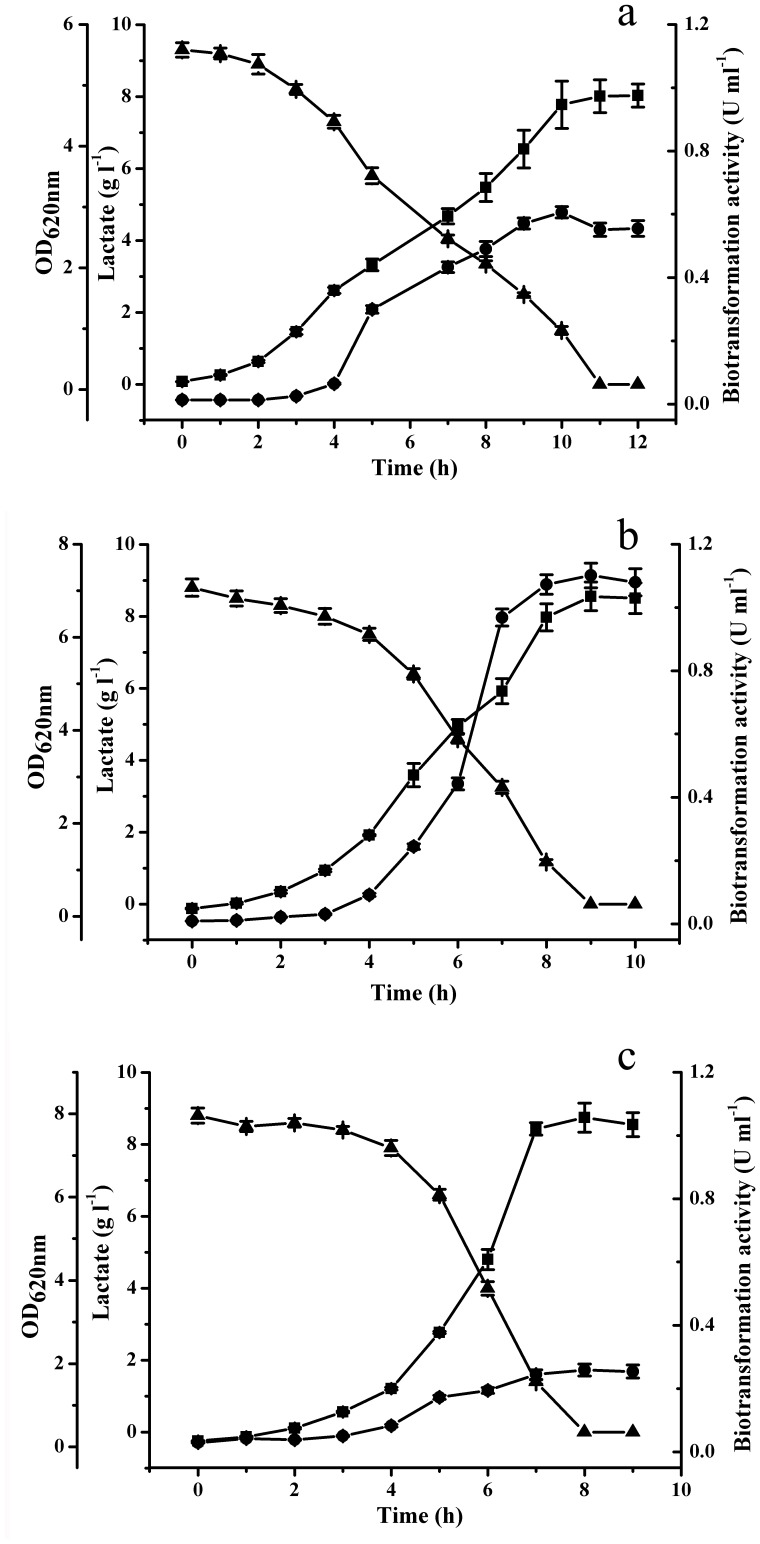
Time course of *P. stutzeri* SDM growth in the media with different dissolved oxygen. (a) 5%; (b) 15%; (c) 30%. (▪) OD_620nm_; (•) Biotransformation activity; (▴) dl-Lactate. Data are the average ± SD of three separate experiments.

In a previous report, *Pseudomonas stutzeri* SDM was reported to have the ability to produce pyruvate from lactate with oxygen as the terminal electron acceptor [8]. No hydrogen peroxide was produced during *P. stutzeri* SDM catalyzed lactate oxidation, which made the strain a promising biocatalyst for the commercial pyruvate production. NAD-independent lactate dehydrogenases (iLDHs) were reported to be involved in the lactate oxidation process [8]–[12]. However, iLDHs could not directly use the oxygen as the electron acceptor, which made the lactate oxidation process in strain SDM rather confusing. To further explore the strain with regard to pyruvate production, the aim of the present study is to determine how iLDHs are involved in the oxidation of lactate. After illustrating the pyruvate-producing mechanism, optimal conditions for the production of pyruvate from a cheap substrate, dl-lactate, by the lactate-utilizing strain SDM were also developed.

## Results

### Pyruvate-producing Mechanism in *P. stutzeri* SDM

*P. stutzeri* SDM possesses 2 inducible iLDHs that made the strain a good biocatalyst for the 2-oxo-carboxylate production [9], [13]–[15]. Unlike lactate oxidase, iLDHs could not oxidize lactate with oxygen as the directly electron acceptor. There should be an electron transfer system between iLDHs and oxygen in the lactate-utilizing strain SDM. To elucidate the electron transfer system in this case, the effects of different electron transfer inhibitors such as diphenylamine, antimycin A, NaN_3_, and salicylhydroxamic acid on the pyruvate-producing activity of the *P. stutzeri* SDM crude extract were studied. As shown in Table 1, the cytochrome *c* reductase inhibitor antimycin A distinctly inhibited the pyruvate-producing activity. This implied that iLDHs in *P. stutzeri* SDM might use quinone as the natural electron acceptor. The electron acquired by quinone would be transferred to cytochrome (by cytochrome *c* reductase) and then terminally transferred to oxygen (by cytochrome oxidase). In addition to the traditionally cytochrome oxidase, *Pseudonomas* strains also have some alternative cytochrome oxidase, such as cytochrome *cbb*_3_ oxidase [16]. Although the cytochrome oxidase inhibitors (diphenylamine, NaN_3_, and salicylhydroxamic acid) exhibited different inhibition effects on the l- and d-lactate oxidation activities of *P. stutzeri* SDM due to the complexity of the cytochrome oxidase system in *Pseudonomas* strains, those results implied the roles of cytochrome in the lactate oxidation process.

**Figure 3:**
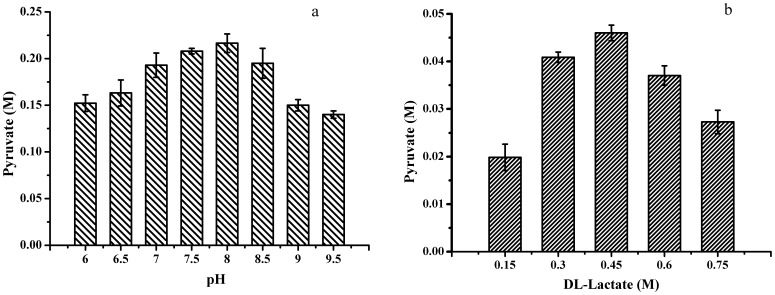
Optimization of biocatalysis conditions. (a) Effect of pH on pyruvate production; (b) Effect of lactate concentrations on pyruvate production.

Next, a cytochrome *c* fraction was purified from *P. stutzeri* SDM (Figure 1a). After oxidizing cytochrome *c* with ferricyanide, a crude extract of *P. stutzeri* SDM and dl-lactate were added. As shown in Figure 1b, the addition of dl-lactate to the reaction system produced a characteristic of reduced cytochrome *c*. The reduction of cytochrome *c* by dl-lactate further supported the participation of cytochrome in the lactate oxidation process. On the basis of the results mentioned above, it is concluded that iLDHs and the electron transport chain account for the observed oxidation of lactate in *P. stutzeri* SDM.

**Figure 4:**
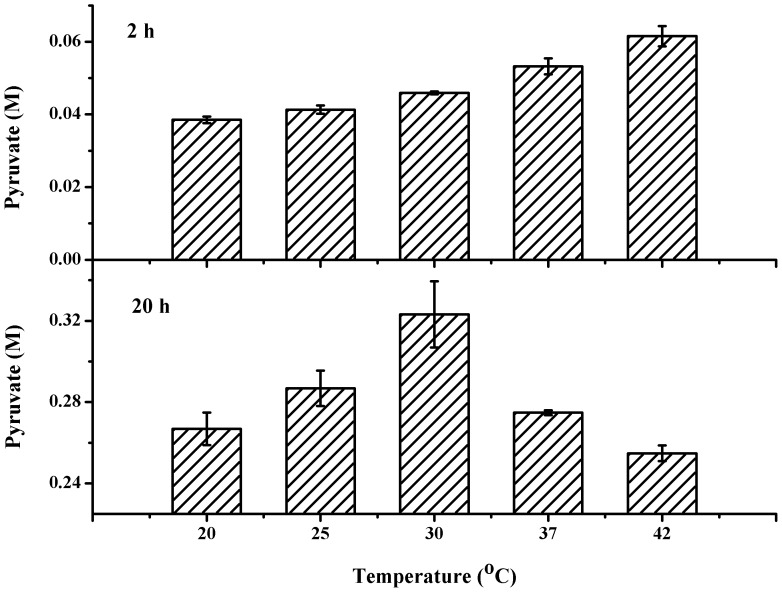
Effect of temperature on the production of pyruvate from dl-lactate.

### Effect of Dissolved Oxygen on Biotransformation Activity

In the case of the lactate oxidation mechanism in *P. stutzeri* SDM, the components of the respiratory chain may influence biotransformation activity distinctly. Because the components of the respiratory chain are different under various dissolved oxygen (DO) concentrations, the effect of DO on the biotransformation activity of *P. stutzeri* SDM was studied in a 5-l reactor. As shown in Figure 2, when 15% DO was employed in the culture, the highest biotransformation activity was obtained.

**Figure 5:**
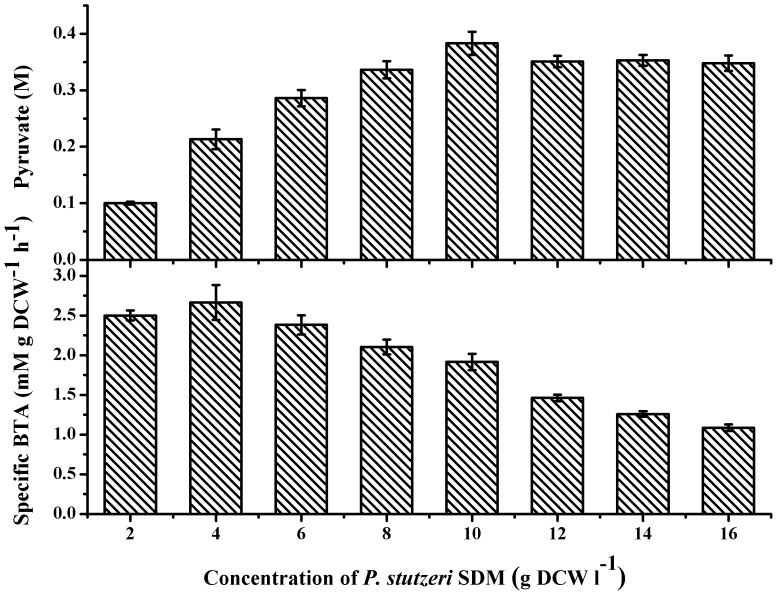
Effect of concentration of *P. stutzeri* SDM on the production of pyruvate from dl-lactate. BTA: biotransformation activity.

d-Lactate or l-lactate has been employed in pyruvate production [1], [5], [7]. Because of the low price and large sources of racemic lactate compared to optical lactate, dl-lactate was used as the substrate for pyruvate production in this work.

**Table 2:** Effect of DO on the pyruvate production.

DO	Pyruvate concentration (M)	Reaction time (h)	Productivity (mM h^−1^)
5%	0.387	39	9.92
15%	0.44	29	15.17
30%	0.186	25	7.44

### Optimal Range of pH

Influence of reaction pH on pyruvate production was determined in 100 mM phosphate buffer containing 6 g dry cell weight (DCW) l^−1^ of cell biomass of *P. stutzeri* SDM and 0.4 M dl-lactate at 30°C. As shown in Figure 3a, after biocatalysis for 24 h, the highest pyruvate production was detected at pH 8.0.

**Figure 6:**
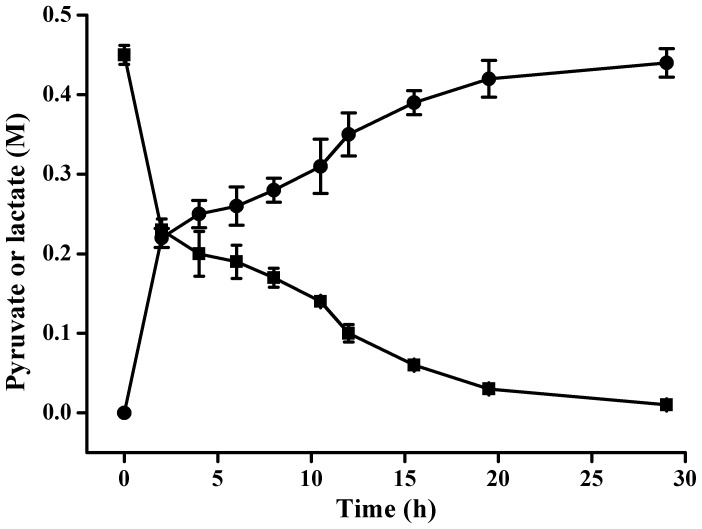
Time course of pyruvate production by *P. stutzeri* SDM. 10 mM EDTA was added in the biocatalysis system to inhibit pyruvate degradation. (▪) Pyruvate; (•) dl-lactate. Data are the average ± SD of three separate experiments.

### Effect of dl-lactate Concentration on Biocatalysis

Substrate concentration was also important for the conversion of DL-lactate to pyruvate. Effect of the concentration of dl-lactate on biocatalysis was investigated to determine its optimal range. The dl-lactate concentration was varied from 0.15 to 0.75 M. As shown in Figure 3b, after 2 h of biocatalysis, the highest pyruvate production was detected when the concentration of dl-lactate was 0.45 M. High DL-lactate concentration (more than 0.6 M) would lead to substrate inhibition and resulted in lower pyruvate concentrations.

**Table 3:** Overview of literature on the enzymatic synthesis of pyruvate from lactate.

Substrate	Biocatalyst	Pyruvate (g l^−1^)	Yield (%)	Reference
l-Lactate	Whole cells of *Acinetobacter* sp. WLIS with lactate oxidase component	44.6	72	17
l-Lactate	Permeabilized cells of *Hansenula Polymorpha* or *Pichia pastoris* expressingboth glycolate oxidase and catalase	111.9	96	29
d-Lactate	*Proteus vulgaris* or *Proteus mirabilis* with HVOR and artificial mediator regeneration system	53.8	94	27, 28
d-Lactate	Whole cells of *Acetobacter* sp. ATCC 21409	10	95	30
dl-Lactate	Whole cells of *P. stutzeri* SDM with iLDHs	22.6	88.6	8
dl-Lactate	Crude extract of *Pseudomonas putida* SM-6 with lactate oxidase and catalase	10.5	82	31
dl-Lactate	Whole cells of *S. marcescens* ZJB-07166	23.1		32
dl-Lactate	Whole cells of *P. stutzeri* SDM with iLDHs	48.4	98	This study

### Optimal Reaction Temperature

To investigate the influence of reaction temperature on pyruvate production, the reaction was carried out at different temperatures at pH 8.0. As shown in Figure 4, the highest pyruvate production was detected at 30°C after 24 h. Although high pyruvate production could be detected at 42°C after 2 h, because of biocatalyst instability under high temperature, the optimal reaction temperature was determined to be 30°C.

### Optimal Range of Cell Biomass

Biocatalysis was carried out with 2–16 g DCW l^–1^ of *P. stutzeri* SDM as the biocatalyst. As shown in Figure 5, 10 g DCW l^–1^ of *P. stutzeri* SDM was optimal and produced the highest pyruvate concentration and relatively high specific biotransformation activity.

### Optimal Range of DO

The biocatalysis production of pyruvate from lactate was a bio-oxidation process. Oxygen would be the terminal electron acceptor. Thus, oxygen was also the substrate of the biocatalysis process and the effect of DO on pyruvate production should be investigated. As shown in Table 2, 0.39 M pyruvate was produced after 39 h with a low amount of DO (5%). Pyruvate production was improved by increasing the DO content to 15%. However, with higher DO (30%), the lactate oxidation decreased, and only 0.19 M pyruvate was produced. This might be due to the substrate inhibition effect of oxygen. The optimal DO content was then determined to be 15%. This result was consistent with our previous report which related to the production of pyruvate from l-lactate [17].

Combining these results, an optimal biotransformation system for the production of pyruvate from dl-lactate was developed. The biocatalysis was conducted at 30°C in distilled water (pH was adjusted to 8.0) with 10 g DCW l^−1^ of *P. stutzeri* SDM as the biocatalyst. The DO saturation was controlled at 15%. The dl-lactate concentration in the 5-l reactor was about 0.45 M. As shown in Figure 6, 0.44 M pyruvate was obtained from 0.45 M dl-lactate after 29 h of biotransformation.

## Discussion

Pyruvate is an important starting material widely applied in chemical, pharmaceutical, and agrochemical industries [6], [18]. Commercial pyruvate is produced by dehydration and decarboxylation of tartaric acid. This classical chemical route is energy-intensive and controversial with issues of environmental protection and sustainable process development [6], [7]. Novel systems which produce pyruvate by biotechnological methods have been the research focus [19]–[22]. Microbial fermentation currently plays a dominant role in biotechnological production of pyruvate [23]–[26]. High concentration (135 g l^−1^) and high volumetric productivity (6 g l^−1^ h^−1^) of pyruvate have been obtained through fermentation [25], [26]. However, biocatalysis processes, due to their simple composition of reaction mixture, high conversion rate of substrate, and convenience of recovery, are also promising in the biotechnological production of pyruvate [6].

Lactate, which can be easily produced from biomass, is the most promising substrate in the biocatalysis production of pyruvate [1], [7]. Of the enzymes employed in the lactate-based pyruvate production, lactate oxidase has been studied extensively [1], [7]. Lactate oxidase catalyzes pyruvate formation from l-lactate with oxygen as the second substrate, giving hydrogen peroxide as a byproduct. Hydrogen peroxide decomposes pyruvate to acetate, carbon dioxide, and water, lowering the production yield. If the problem of further oxidation of pyruvate by hydrogen peroxide can be solved, the biocatalysis production of pyruvate from lactate has the potential to be commercialized because of the low price of lactate [1], [6], [7]. *P. stutzeri* SDM was confirmed to have the ability to produce pyruvate from lactate with the merit of no hydrogen peroxide production. iLDHs in *P. stutzeri* SDM firstly acquired the electron from the lactate and then the electron would be terminally transferred to oxygen. However, the electron transfer process has never been clarified in previous works.

Pyruvate production by (2*R*)-hydroxycarboxylate-viologen-oxidoreductase (HVOR) in *Proteus vulgaris* or *Proteus mirabilis* with the addition of artificial redox mediators was studied in previous reports [27], [28]. The regeneration of artificial redox mediators was achieved by chemical or electrochemical methods. However, in the case of *P. stutzeri* SDM, pyruvate was produced without the addition of an artificial redox mediator. The reduction of cytochrome *c* in *P. stutzeri* SDM by dl-lactate implied that the electron transfer components of respiratory chain might play an important role in lactate oxidation. Effects of different respiratory chain inhibitors on the pyruvate production further identified the involvement of the electron transfer chain in the lactate oxidation process. Unlike *Proteus* strains, the cofactor regeneration of iLDHs in *P. stutzeri* SDM may employ the inherent electron transfer system of the strain. This process, which excludes the expensive cofactor regeneration step, makes *P. stutzeri* SDM a rather practicable alternative for the biocatalytic production of pyruvate. Under the optimal conditions, with biocatalyst prepared from 10 g DCW l^−1^ of *P. stutzeri* SDM, 0.44 M pyruvate was obtained after 29 h.

As shown in Table 3, pyruvate production from d-lactate, l-lactate, and dl-lactate has been studied in previous works [29]–[32]. Genetically modified *Hansenula polymorpha* and *Pichia pastoris* cells expressing glycolate oxidase could catalyze l-lactate into pyruvate with rather high yield [29]. For the decomposition of the byproduct hydrogen peroxide, co-expression of catalase with glycolate oxidase in genetically modified yeasts was needed [1], [7]. Whole cells of *P. stutzeri* SDM catalyzed lactate oxidation without the production of hydrogen peroxide. On the other hand, the glycolate oxidase could only utilize l-lactate as the substrate but a large amount of lactate produced today is a racemic mixture of both stereospecific forms. *P. stutzeri* SDM possesses 2 inducible iLDHs which give the strain the ability to use dl-lactate, a much cheaper substrate than the optical lactate, as the substrate to produce pyruvate.

Recently, *Serratia marcescens* ZJB-07166 has been applied in the biotransformation of dl-lactate to pyruvate. The pyruvate concentration of 0.21 M was achieved under an optimum condition [32]. The newly isolated *S. marcescens* ZJB-07166 was regarded as a promising strain for pyruvate production at an industrial scale [32]. Under the conditions optimized in the present study, a much higher concentration of pyruvate (0.44 M) was obtained from the cheap substrate dl-lactate than in previous reports.

In conclusion, the lactate-utilizing *P. stutzeri* strain SDM was confirmed to catalyze lactate oxidation through iLDHs and the inherent electron transfer chain. Preparation of pyruvate from dl-lactate was carried out under the following optimal conditions: DCW, 10 g l^−1^; pH 8.0; temperature, 30°C; DO saturation, 15%; and dl-lactate concentration, 0.45 M. After 29 h of biotransformation, pyruvate was obtained at a high concentration (48.4 g l^−1^) and a high yield (98%). The biocatalysis process introduced in this study provides not only a rather feasible method for pyruvate production but also a green pathway for the utilization of lactate produced from biomass.

## Materials and Methods

### Chemicals

l-Lactate and bovine serum albumin were purchased from Sigma. d-Lactate was purchased from Fluka. dl-Lactate was purchased from Wujiang Ciyun Flavor & Fragrance Co. Ltd. (P. R. China). Diphenylamine, antimycin A, and salicylhydroxamic acid were purchased from Sigma. All other chemicals were of reagent grade.

### Microorganism and Biocatalyst Preparation

*P.* stutzeri SDM (China Center for Type Culture Collection No. M 206010) isolated from soil was used [9]. The minimal salt medium (MSM) supplemented with 10.0 g 1^−1^ dl-lactate was used as the fermentation medium [9]. For biocatalyst preparation, cells of the SDM strain cultivated at 30°C were harvested from MSM by centrifugation, washed twice with 0.85% (w/v) sterile salt water, and then resuspended in various concentrations with distilled water.

### Preparation of Crude Cell Extract

Cells of the SDM strain grown in MSM containing dl-lactate as the sole carbon source were resuspended in 50 mM Tris-HCl (pH 8.0) and disrupted by sonication (Sonics 500 W/20 KHz, USA) in an ice bath. The disrupted cells were subjected to centrifugation for 20 min at 12,000 *g*, and the supernatant was used as crude cell extract.

### Purification of Cytochrome in *P. stutzeri* SDM

The membrane fraction of *P. stutzeri* SDM was prepared from the crude cell extract by centrifugation at 147,000 *g* for 180 min. Triton X-100 (10%, w/v) was added to the membrane fraction to a final concentration of 1 mg mg^−1^ of protein. The supernatant (detergent extract) was applied to a column of DEAE Sepharose Fast Flow equilibrated with Buffer A: 50 mM Tris-HCl (pH 8.0) containing 1 mM dithiothreitol (DTT), 5 mM MgSO_4_, 0.1% Triton X-100, and 1 mM EDTA. The column was washed with Buffer B: 50 mM Tris-HCl (pH 8.0) containing 1 mM DTT, 200 mM KCl, 5 mM MgSO_4_, 0.1% Triton X-100, and 1 mM EDTA at a flow rate of 5 ml min^−1^. Cytochrome from column effluents was monitored by measuring the absorbance at 410 nm. The fractions containing cytochrome were concentrated by ultrafiltration and desalted with gel G-25. The cytochrome pool after desalting was then applied to a column of DEAE A-25 pre-equilibrated with Buffer A. The column was washed with a linear gradient of 0–100% Buffer B at a flow rate of 0.5 ml min^−1^. The fractions containing cytochrome were concentrated and stored at 0 to 4°C. Native polyacrylamide gel electrophoresis (native-PAGE) was performed on a 5–12% native polyacrylamide gradient gel with a Mini-Protean III system (Bio-Rad) according to the protocol by Davis [33]. After electrophoresis, the gel was stained for the protein with Coomassie Brilliant Blue R-250.

### Pyruvate-producing Activity Assay

Pyruvate-producing activity was determined in 1 ml of 67 mM phosphate buffer (pH 7.4) containing 20 mM l-lactate or d-lactate. The reaction was initiated by the addition of whole cells or crude cell extract of SDM at 37°C for 10 min. The reaction was stopped by adding 0.05 ml of 1 M NaOH. The mixture was subjected to centrifugation for 1 min at 16,000 *g*, and a 0.5-ml portion of the supernatant was used for pyruvate detection. One unit was defined as the amount of enzyme that converted lactate to 1.0 µM pyruvate per minute under the test conditions.

### Analytical Methods

Accurate concentrations of lactate and pyruvate were analyzed by HPLC (Agilent 1100 series, Hewlett-Packard, USA) using an Aminex HPX-87H column (Bio-Rad), which was running at 0.4 ml min^−1^ with 10 mM H_2_SO_4_ as eluent at 55°C [34]. UV and visible absorption spectra of the cytochrome were measured by an Ultrospec™ 2100 *pro* UV/visible spectrophotometer (GE Healthcare). Protein was determined by the Markwell variation of the Lowry method, with bovine serum albumin as the standard [35].
